# Associations between Socio-Economic Status and Unfavorable Social Indicators of Child Wellbeing; a Neighbourhood Level Data Design

**DOI:** 10.3390/ijerph182312661

**Published:** 2021-12-01

**Authors:** Minke R. C. van Minde, Marlou L. A. de Kroon, Meertien K. Sijpkens, Hein Raat, Eric A. P. Steegers, Loes C. M. Bertens

**Affiliations:** 1 Department of Obstetrics & Gynaecology, Division of Obstetrics and Prenatal Medicine, Erasmus University Medical Centre, 3015 CN Rotterdam, The Netherlands; m.dekroon@erasmusmc.nl (M.L.A.d.K.); m.sijpkens@erasmusmc.nl (M.K.S.); e.a.p.steegers@erasmusmc.nl (E.A.P.S.); l.bertens@erasmusmc.nl (L.C.M.B.); 2 Department of Public Health, Erasmus University Medical Centre, 3015 CN Rotterdam, The Netherlands; h.raat@erasmusmc.nl; 3 Department of Health Sciences, University Medical Centre, 9713 GZ Groningen, The Netherlands

**Keywords:** socio-economic status, deprived neighbourhoods, child wellbeing, families on welfare, unemployed youth, delinquent youth

## Abstract

Background: Living in deprivation is related to ill health. Differences in health outcomes between neighbourhoods may be attributed to neighbourhood socio-economic status (SES). Additional to differences in health, neighbourhood differences in child wellbeing could also be attributed to neighbourhood SES. Therefore, we aimed to investigate the association between neighbourhood deprivation, and social indicators of child wellbeing. Methods: Aggregated data from 3565 neighbourhoods in 390 municipalities in the Netherlands were eligible for analysis. Neighbourhood SES scores and neighbourhood data on social indicators of child wellbeing were used to perform repeated measurements, with one year measurement intervals, over a period of 11 years. Linear mixed models were used to estimate the associations between SES score and the proportion of unfavorable social indicators of child wellbeing. Results: After adjustment for year, population size, and clustering within neighbourhoods and within a municipality, neighbourhood SES was inversely associated with the proportion of ‘children living in families on welfare’ (estimates with two cubic splines: −3.59 [CI: −3.99; −3.19], and −3.00 [CI: −3.33; −2.67]), ‘delinquent youth’ (estimate −0.26 [CI: −0.30; −0.23]) and ‘unemployed youth’ (estimates with four cubic splines: −0.41 [CI: −0.57; −0.25], −0.58 [CI: −0.73; −0.43], −1.35 [−1.70; −1.01], and −0.96 [1.24; −0.70]). Conclusions: In this study using repeated measurements, a lower neighbourhood SES was significantly associated with a higher prevalence of unfavorable social indicators of child wellbeing. This contributes to the body of evidence that neighbourhood SES is strongly related to child health and a child’s ability to reach its full potential in later life. Future studies should consist of larger longitudinal datasets, potentially across countries, and should attempt to take the interpersonal variation into account with more individual-level data on SES and outcomes.

## 1. Background

Deprivation is defined as “a state of observable and demonstrable disadvantage, relative to the local community or the wider society or nation to which an individual, family or group belongs” [1]. Socio-economic status (SES) refers to an individual’s level of resource or prestige in relation to others and is traditionally measured through factors such as wealth/income, place on a social hierarchy or class system, and level of education or occupation [2]. SES may be assessed at the individual or contextual level, e.g., neighbourhood level [3]. For the remainder of this work, we will focus on the contextual level SES, where low SES can be understood as indicative of material, financial or social deprivation in a neighbourhood.

Children rely on their parents SES, such as the neighbourhood they live in and the financial status of the family they belong to [4,5,6]. The effects of neighbourhood SES on babies and children have been studied in previous literature, for instance, when a pregnant woman lives in a deprived neighbourhood, she has higher odds of adverse perinatal outcomes, such as preterm birth (<37 weeks gestational age), a child born small for gestational age (birthweight < 10th percentile) and stillbirth [7]. Moreover, children living in deprivation are more likely to be overweight or obese during childhood and to have developmental delay when growing up [8,9,10]. Growing up in deprivation is related to higher odds of smoking and less physical activity in later life [11]. The longer the exposure to deprivation during childhood, the higher the odds of developmental delay and deviant behaviour in adolescence [8,9]. For adolescents, living in deprivation is associated with less physical activity, and behavioural and psychosocial problems [5,6,12]. As young adults, these children show weaker work commitment [13].

In the past two decades, it has been generally acknowledged that SES operates at multiple levels (e.g., contextual and personal) to affect wellbeing [14,15,16,17]. Contextual level SES measurements, such as neighbourhood SES, have been recognised to provide information about exposures to violence and hazards, as well as access to recreational and institutional resources [14]. For children, there is evidence that neighbourhood of residence is associated with health, school achievement and behavioural outcomes, even when individual level income and education of the parent are controlled for [14,17,18,19]. Chetty et al. (2017) showed that neighbourhoods in which children grow up shape their earnings, college attendance rates and their fertility in later life [20]. There is also evidence that living in a low-SES neighbourhood may contribute to the development of behavioural problems and increase the likelihood of single parenthood and teenage motherhood [21]. Additionally, teenage motherhood is often accompanied with poor educational achievement and unemployment of the mother [21]. Osofsky argued that children growing up in poor urban environments are frequently exposed to guns, knives, drugs, and acts of random violence [22]. Exposure to such violence also interrupts a child’s ability to solve problems [14]. Furthermore, according to Leventhal and Brooks-Gunn (2000), the most consistent finding is that living in a high-SES neighbourhood has positive benefits for school readiness and school achievement [17].

In the Netherlands, a Western European, developed country, geographical differences in health outcomes between neighbourhoods are high [23,24,25]. These differences may be attributed to neighbourhood SES [20,23,26]. Additional to differences in health, neighbourhood differences in child wellbeing could also be attributed to neighbourhood SES [27,28,29]. To the best of our knowledge, a study on neighbourhood SES and neighbourhood social indicators of child wellbeing has not been conducted before. Most studies only focused on child development instead of wellbeing and on the cross-sectional association between SES and health-related outcomes, while the exposure (SES) and the outcomes, as well as their associations, are not fixed over time [30,31]. Therefore, we aimed to investigate the association between neighbourhood deprivation, based on SES, and social indicators of child wellbeing over a period of 11 years. We used repeated measurements to take into account the changes over time in both SES and child wellbeing indicators. We hypothesised that neighbourhood deprivation affects neighbourhood social indicators of child wellbeing negatively.

## 2. Methods

### 2.1. Study Design and Population

This study uses a neighbourhood-level data design, whereas ecological variables derived from neighbourhoods were used for the analysis. No individual-level data were used for this study, hence the authors did not have access to individual-level data throughout the study. Neighbourhood-level SES scores and neighbourhood-level data on social indicators of child wellbeing from 2005 until 2015 were used to perform repeated measurements. In this paper a neighbourhood is defined as a four digit postal code (PC4) area. Data from 3565 neighbourhoods representing 390 municipalities were initially eligible for analysis, which represent all of the neighbourhoods and municipalities in the Netherlands in 2015.

### 2.2. Social Indicators of Child Wellbeing

Data on social indicators of child wellbeing were provided by ‘Defence for Children’ (www.defenceforchildren.nl, accessed on 28 September 2021), a non-governmental Coalition for Children’s Rights. This coalition monitors data on child wellbeing, and is based on ‘Kid’s Count’, a method adopted from the USA [32]. Neighbourhood-level aggregated data were provided on the proportion of children who were exposed to the unfavourable social indicators of child wellbeing. Data were provided per year from 2005 up to and including 2015 [33]. Not all outcome measures were available for the full study period. Table 1 represents the main and secondary outcome measures. Social indicators available for the full period of eleven years (2005–2015) were selected as main outcome measures, social indicators available less than eleven years were included as the secondary outcome measures.

### 2.3. Socio-Economic Status

The Netherlands Institute of Social Research (SCP) publishes a SES score by PC4 (neighbourhood), every four years. The SCP is a governmental agency, which conducts research into the social aspects of all areas of governmental policy. This SES score indicates the social status in a neighbourhood, compared to other neighbourhoods. The SES score of a neighbourhood is calculated according to characteristics of its inhabitants: education, income and their position in the labour market. A high score represents a high neighbourhood SES, a low score represents a low neighbourhood SES. The average SES score is around 0, with a standard deviation of 1 [34]. Between 1998 and 2014, the overall social status in the Netherlands increased, but in 2016 it decreased. The SCP does not calculate a SES score for neighbourhoods with less than 100 households (0.2% of all Dutch neighbourhoods) [34]. Because the SES score is calculated by the SCP every four years, the SES score of 2002 was assigned to the year 2005, the SES score of 2006 was assigned to the years 2006–2009, the SES score of 2010 was assigned to the years 2010–2013 and the SES score of 2014 was assigned to the years 2014–2015, respectively.

### 2.4. Statistical Analyses

Descriptive statistics were applied to calculate the median and 95% ranges for the SES scores and the outcome measures. Plots were created to depict the different social indicators of child wellbeing and explore their trends over time. Neighbourhoods with one or more missing SES scores were excluded (*n* = 7). Outliers in the SES score were removed to better approximate a normal distribution of the data. Hence, the lowest 2.5% of SES scores were removed, after which 3531 neighbourhoods (99% of all Dutch neighbourhoods) embedded in all 390 municipalities remained for the analyses. Separate plots were created to assess the linearity of the relationship between the social indicators of child wellbeing and SES score. For the repeated measurements, linear mixed models (LMM) with random intercepts were used to estimate the association of SES score (continuous measure) and the prevalence of social indicators for children (continuous measure), with neighbourhood as analyses-unit. Cubic splines were applied to the SES score when there were non-linear relationships between SES score and the main or secondary outcomes. The number of used knots needed differed per outcome measure (range: 2–7). Maximum-Likelihood Estimation (MLE) was applied to estimate model parameters. Two-level hierarchical random-intercept models with neighbourhoods at level one, nested within municipalities at level two were specified. This allows for the incorporation of both neighbourhood-level and municipality-level characteristics, as well as the adjustment for clustering within a neighbourhood itself and for clustering of neighbourhoods within a municipality. The variance estimates of the random effects and the beta estimates of the fixed effects were reported, with corresponding 95% confidence intervals.

A generalised linear regression analysis was used for all outcome measures. The model used was a function of SES and year, in which both the independent variable as well as the interaction with SES was added. Additionally, a random intercept for neighborhood and a nested random effect for municipality was added.

### 2.5. Sensitivity Analysis and Subgroup Analysis

Sensitivity analyses were performed to assess whether weighing for population size per neighbourhood resulted in a better fit of the LMM’s. The analyses demonstrated a better fit when this weighing was applied and was included into the models. Finally, a subgroup analysis was performed in children of the age group zero up to and including two years old. For this subgroup, only five outcome measures were available for only one year. The available outcome measures were: ‘reported and confirmed child abuse (2014)’, ‘children living in families on welfare (2015)’, ‘child social services involved (2015)’, ‘single parents (2015)’ and ‘children with a handicap (2015)’. Generalised linear regression analyses were applied for the subgroup and the whole group (0–17 year old children) as a comparison. For all analyses, the significance was set at alpha <0.05, two tailed. Analyses were performed using R studio version 1.0.153 (R studio) and, specifically, the LME4 package was used for the LMM.

## 3. Results

**Figure 1:**
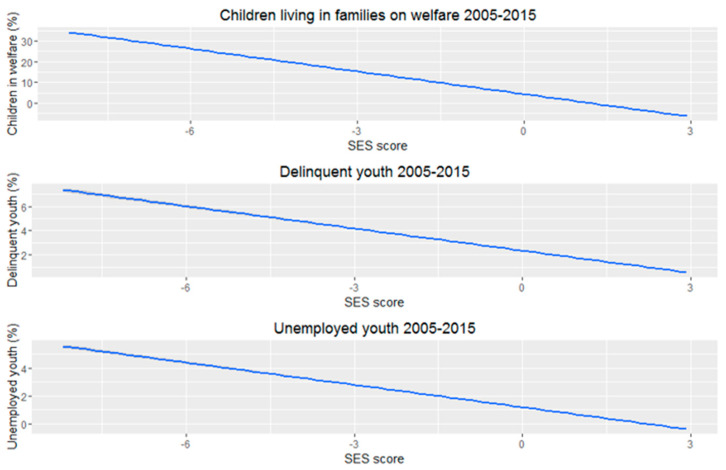
Trends of children living in families on welfare, delinquent youth and unemployed youth over time. (2005–2015).

Aggregated data of 3558 neighbourhoods was available. Table 2 features the characteristics of this dataset. Afterwards, SES score outliers (the lowest 2.5%) were removed, after which 3531 (99%) neighbourhoods distributed over 390 (100%) municipalities, remained for the final analyses. Figure 1 illustrates the increasing average prevalence of ‘children living in families on welfare’ over time, and the decreasing average proportion of ‘delinquent youth’ and ‘unemployed youth’ over time.

Table 3 displays the mixed models for the main outcome measures after applying weights for population size. The results of the initial analyses without applying weights are presented in Supplementary Table S1. The analyses of the main outcomes show that neighbourhood SES is inversely related with the prevalence of unfavourable social indicators of child wellbeing. All associations between neighbourhood SES and the outcomes follow different curves/shapes; ‘Children living in families on welfare’ shows an inverted exponential association, (with a steeper slope for lower SES scores), a more linear association for ‘delinquent youth’, and an inverted sigmoid association for ‘unemployed youth’ (with a steeper slope for medium SES scores).

The results of the secondary outcome measures are presented in Supplementary Table S2. The SES score showed an almost linear association with ‘child social services involved’, and an inverted sigmoid association with ‘teenage mothers’, ‘children living with a single parent’ and ‘school drop-outs’(with steeper slopes for higher SES scores. There is an inverted exponential association with ‘reported and confirmed child abuse’, ‘children with a handicap’ and ‘children in special education’ (with steeper slopes for lower SES scores). The association of SES score with ‘disadvantaged pupils’ represents an undefinable shape. Additionally, higher SES scores were significantly associated with higher proportions of ‘children participating in sports associations’ in a neighbourhood, showing an exponential relationship.

The characteristics of the subgroup of children in the age group zero until two years old are presented in Supplementary Table S3. The linear regression analyses indicated an inverse association between SES score and ‘reported and confirmed child abuse (2014)’, ‘children living in families on welfare (2015)’, ‘child social services involved (2015)’ and ‘single parents (2015)’. Similar associations were found in the linear regression analyses for the total population of 0–17 year old children in the equivalent years (Supplementary Table S4).

## 4. Discussion

We investigated the association between neighbourhood SES and social indicators of child wellbeing using repeated measurements with a one year measurement interval over a period of 11 years. Our results indicate that a lower neighbourhood SES was significantly associated with a higher prevalence of unfavourable social indicators of child wellbeing. Our findings indicate that low neighbourhood SES scores are strongly associated with higher proportions of children with ‘unfavorable’ social indicators in a neighbourhood. The steeper slopes for lower SES scores indicate that these findings are even more pronounced for neighbourhoods with the lowest SES scores.

For the main outcome measure ‘children living in families on welfare’, the explained variance was 0.9, indicating that this outcome is very closely related to the neighbourhood SES scores. Furthermore, for almost all outcome measures significant associations were found, with explained variances varying between 0.44 and 0.97. This may be due to different municipal or governmental policies regarding child social services for younger and older children, for instance, with a stronger focus on social services for pregnant women and infants in low SES neighbourhoods [35]. Our data did not show a significant relationship between SES score and the secondary outcome measure ‘disadvantaged pupils’ (2005–2012).

Our findings are consistent with previous literature from other countries (e.g., Great Britain and United States of America) and emphasize the relationship between neighbourhood SES and unfavourable social indicators during childhood. Neighbourhood SES is often considered as a constant variable. With our methodology and by using repeated measurements, we took the possible variety in SES into consideration to measure our outcome. Our study results confirmed that a low neighbourhood SES is associated with unfavourable social indicators of child wellbeing such as child abuse, living with a single parent, delinquency, less sports participation, and teenage motherhood. The relevance of this finding is illustrated by the fact that these social indicators are related to the problem solving ability and adaptive learning of the child and unemployment and social isolation of the parent(s) [14,21].

Chetty et al. (2017) argued that neighbourhoods affect a child’s long-term outcomes through childhood exposure effects. The outcomes of people who move into a certain neighbourhood are likely to converge with those of permanent residents in the destination to which they move. The longer a child lives in a certain neighbourhood, the stronger the neighbourhood effects on their health related outcomes [20]. This implies that neighbourhood indicators influence the outcomes of its residents, rather than its inhabitants determine neighbourhood SES.

Children living in families on welfare grow up in an environment where parents, neighbours and other family members need to make an effort to get by. Leisure activities and sports are luxuries those children do not have access to. If those activities are not enabled through neighbourhood services or by the municipality, these children will lag behind on their peers. Delinquent and unemployed youth who live in an environment where careers are not supported or encouraged will negatively influence each other and their peers [32]. Our individual outcome measures are all influenced by different socio-economic stressors, and thereby show different slopes.

### Strengths and Limitations

A major strength of our study is that the vast majority of neighbourhoods (99%) in the Netherlands were included in the analyses. Additionally, we were able to use data over a period of 11 years, enabling us to use repeated measurements and create a more robust estimation of the associations while taking variations over time into account. Similar associations were found in our sensitivity and subgroup analyses, indicating that our findings are robust. A limitation of this study is the absence of personal-level data. With personal-level data it would have been possible to create a three-level model, taking the interpersonal variation of people living in a neighbourhood into account. Another limitation of this study is that we were not able to analyse the effects of time trends in SES on health and wellbeing of its residents. It is hypothesised that, for instance, an increasing neighbourhood SES benefits the health of its residents. It is likely that this effect is delayed, showing a so-called lagging effect on health outcomes [36]. In order to assess the effects of socio-economic trends over time, larger longitudinal datasets are needed, including data from multiple decades of time, and personal level data, including data on residents moving into and out of a certain neighbourhood [20,36]. In our literature search, we did not find any studies showing no relationship between SES and the wellbeing of children and youth. In conclusion, future studies should consist of larger longitudinal datasets and should attempt to take the interpersonal variation into account. For example, a longitudinal study into successful and unsuccessful policies and implementation processes of help- and care facilities. When it comes to individual data studies, a qualitative semi-structured interview approach combined with a qualitative/quantitative survey could be considered.

## 5. Conclusions

This study underlines the relationship between a low neighbourhood SES and a high proportion of children with unfavourable social indicators of child wellbeing in a neighbourhood, including stronger effects for lower SES scores. This contributes to the body of evidence that neighbourhood SES is an important factor related to health and social indicators of child and adolescent wellbeing and wellbeing in later life. The general ecological hypothesis states that as the number of stressors (i.e., social disorder, environmental deterioration, violence, and crime) in a neighbourhood rise, distress among those living in the neighbourhood increases [37,38,39]. A high proportion of unfavourable social indicators of child wellbeing in a neighbourhood could be a result of these stressors, and contributes to widening of the gap between people of different socio-economic status. Although neighbourhood SES is largely driven by the characteristics of its adult inhabitants, it also affects a child’s ability to develop to its full potential, which renders inequality between children growing up in low or high SES neighbourhoods. Attention must be paid to these inequalities, specifically in children, by governmental, social, and healthcare institutions, in order to provide equal opportunities for all.

## Figures and Tables

**Table 1:** Social indicators of child wellbeing by ‘Defence for Children’.

Main Outcomes		
Social Indicator of Child Wellbeing	Definition by ‘Defence for Children’	Years Available
Children living in families on welfare	The number of children (age group 0–17) living in families on welfare per neighbourhood, divided by the total number of children in the age group living in that neighbourhood.	2005–2015
Delinquent youth	The number of delinquent adolescents (age group 12–21 years) per neighbourhood, divided by the total number of adolescents in the age group living in that neighbourhood.	2005–2015
Unemployed youth	The number of adolescents (age group 16–22 years) who are not working and looking for a job per neighbourhood, divided by the total number of adolescents in the age group living in that neighbourhood.	2005–2015
**Secondary Outcomes**		
**Social Indicator of Child Wellbeing**	**Definition by ‘Defence for Children’**	**Years Available**
Child social services involved	The number of children (age group 0–17) where child social services is involved (i.e., foster care, youth care or child protection services) per neighbourhood, divided by the total number of children in the age group living in that neighbourhood.	2013–2015
Teenage mothers	The number of teenage mothers (age group 15–19) per neighbourhood, divided by the total number of children in the age group living in that neighbourhood.	2005–2012
Single parents	The number of children (age group 0–17) who have a single parent per neighbourhood, divided by the total number of children in the age group living in that neighbourhood.	2013–2015
Reported and confirmed child abuse	The number of children (age group 0–17), where child abuse was reported and confirmed per neighbourhood, divided by the total number of children in the age group living in that neighbourhood.	2005–2014
Children with a handicap	The number of children with a handicap (age group 0–17) per neighbourhood, divided by the total number of children in the age group living in that neighbourhood.	2012–2015
Disadvantaged pupils	The number of disadvantaged pupils (in primary education) per neighbourhood, divided by the total number of children in primary education living in that neighbourhood.	2005–2012
Children in special education	The number of children in special education (in primary and secondary education) per neighbourhood, divided by the total number of children in primary and secondary education living in that neighbourhood.	2013–2015
Children participating in sport clubs	The number of children (age group 0–17) who are participating in a sports association per neighbourhood, divided by the total number of children in the age group living in that neighbourhood.	2014–2015

**Table 2:** Characteristics of social indicators of child wellbeing.

Variable	Mean	Median	95% Range	Min–Max	Missing (%)
SES score	0.046	0.21	−2.63–1.83	−8.19–2.93	0.0
Children living in families on welfare (%)	4.2	2.1	0.0–22.9	0.0–62.5	0.2
Delinquent youth (%)	2.3	1.8	0.0–7.7	0.0–50.0	0.2
Unemployed youth (%)	1.2	0.6	0.0–5.8	0.0–19.2	0.2
Child social services involved (%)	10.2	10.3	4.0–15.0	0.0–51.6	0.0
Teenage mothers (%)	0.6	0.2	0.0–3.1	0.0–11.1	0.2
Single parents (%)	12.0	11.0	0.0–33.3	0.0–37.5	0.0
Reported and confirmed child abuse (%)	0.6	0.4	0.0–2.9	0.0–13.3	0.2
Children with a handicap (%)	2.3	2.2	0.0–5.0	0.0–37.5	0.1
School drop-outs (%)	2.9	2.5	0.0–8.2	0.0–40.0	0.2
Disadvantaged pupils (%)	14.9	10.8	0.0–58.2	0.0–98.2	0.2
Children in special education (%)	2.2	1.9	0.0–5.9	0.0–57.1	0.0
Children participating in sport clubs (%)	43.2	43.8	17.4–66.2	0.0–93.3	0.0

*n* = 3558, neighbourhoods with missing SES scores (*n* = 7) were excluded. Data is presented as mean, median score, 95% range, minimum and maximum and percentage of missing data.

**Table 3:** Results of the main outcome measures, weighted for the number of children or adolescents per neighbourhood (*n* = 3531).

Social Indicator of Child Wellbeing	Effect	Beta Estimate (95% Confidence Interval)	Variance Estimate of PC4 Nested within Municipality (SD)	Variance Estimate of Municipality (SD)
Children living in families on welfare	Intercept	4.07 (3.72; 4.42)	17.44 (4.18)	4.35 (2.09)
	SES score, 1	−3.59 (−3.99;−3.19)		
	SES score, 2	−3.00 (−3.33; −2.67)		
	Year	0.047 (0.042; 0.053)		
	Population size (ages 0–17)	0.06 (0.05; 0.07)		
Delinquent youth	Intercept	2.01 (1.92; 2.11)	1.58 (1.26)	0.40 (0.64)
	SES score	−0.26(−0.30; −0.23)		
	Year	−0.179 (−0.184; −0.174)		
	Population size (ages 12–21)	−0.02 (−0.01; 0.03)		
Unemployed youth	Intercept	1.24 (1.07; 1.42)	0.40 (0.63)	0.28 (0.53)
	SES score, 1	−0.41 (−0.57; −0.25)		
	SES score, 2	−0.58 (−0.73; −0.43)		
	SES score, 3	−1.35 (−1.70; −1.01)		
	SES score, 4	−0.96 (−1.24; −0.70)		
	Year	−0.104 (−0.109; −0.10)		
	Population size (ages 16–22)	0.075 (0.067; 0.084)		

SES score 1: first cubic spline, SES score 2: second cubic spline, SES score 3: third cubic spline, etc.

## Data Availability

The data will not be shared, in accordance with the contract with ‘Defense for Children’. The data on social economic status (SCP, www.scp.nl, accessed on 28 September 2021) are publicly available. Data can be provided by the Erasmus MC upon request.

## Supplementary Materials

The following are available online at https://www.mdpi.com/article/10.3390/ijerph182312661/s1, Table S1: Primary analysis for the main outcome measures, estimates of SES score and social indicators of child wellbeing, *n* = 3531; Table S2: Secondary outcome measures, adjusted for the number of children or adolescents living in a certain neighbourhood, *n* = 3531; Table S3: Characteristics of children in the age group 0–2 years old, *n* = 3541; Table S4: Linear regression analyses for SES score and social indicators of child wellbeing, adjusted for the number of children living in a certain neighbourhood, *n* = 3541.

## Abbreviations

LMM	linear mixed models
MLE	Maximum-Likelihood Estimation
PC4	Four digit postal code
SCP	The Netherlands Institute of Social Research
SES	Socio-economic status
