# Supplementary material for: Associations between Socio-Economic Status and Unfavorable Social Indicators of Child Wellbeing; a Neighbourhood Level Data Design
Source: Int J Environ Res Public Health. 2021 Dec 1;18(23):12661. doi: 10.3390/ijerph182312661 (PMC8657207; doi:10.3390/ijerph182312661)
Supplement: Supplementary file 1 [file ijerph-18-12661-s001.zip › ijerph-1421592-supplementary.pdf]

Supplementary Table S1. Primary analysis for the main outcome measures, estimates of SES score and social indicators of child wellbeing, n = 3,531.

**Social indicator of child wellbeing**	**Effect**	**Beta estimate (95% confidence interval)**	**Variance estimate of PC4:municipality (SD)**	**Variance estimate of municipality (SD)**	**R squared**
Children living in families on welfare	Intercept	4.63 (4.29 ; 4.98)	18.24 (4.27)	4.86 (2.20)	0.90
	SES score, 1	-3.43 (-3.83 ; -3.04)			
	SES score, 2	-2.79 (-3.11 ; -2.46)			
	Year	0.045 (0.040 ; 0.050)			
Delinquent youth	Intercept	2.12 (2.04 ; 2.21)	1.58 (1.26)	0.44 (0.67)	0.54
	SES score	-0.27 (-0.31 ; -0.23)			
	Year	-0.18 (-0.183 ; -0.174)			
Unemployed youth	Intercept	1.51 (1.34 ; 1.69)	0.45 (0.67)	0.30 (0.55)	0.33
	SES score, 1	-0.36 (-0.53 ; -0.20)			
	SES score, 2	-0.62 (-0.78 ; -0.47)			
	SES score, 3	-1.44 (-1.79 ; -1.08)			
	SES score, 4	-1.20 (-1.48 ; -0.93)			
	Year	-0.102 (-0.107 ; -0.098)			

Supplementary Table S2. Results of the secondary outcome measures, adjusted for the number of children or adolescents living in a certain neighbourhood, n = 3,531.

**Child social determinant**	**Effect**	**Estimate (95% confidence interval)**	**Variance estimate of PC4 nested within municipality (SD)**	**Variance estimate of municipality (SD)**
	Intercept	14.92 (14.41 ; 15.06)	0.00 (0.00)	2.19 (1.48)
	SES score, 1	-1.74 (-2.13 ; -1.35)		

Child social services involved	SES score, 2	-1.71 (-2.00 ; -1.43)		
	Year	-1.05 (-1.10 ; -1.01)		
	Population size (ages 0-17)	0.017 (0.013 ; 0.023)		
Teenage mothers	Intercept	1.04 (0.0.94 ; 1.13)	0.14 (0.38)	0.06 (0.25)
	SES score, 1	-0.47 (-0.54 ; -0.40)		
	SES score, 2	-1.38 (-1.58 ; -1.18)		
	SES score, 3	-0.75 (-0.89 ; -0.60)		
	Year	-0.035 (-0.039 ; -0.030)		
	Population size (ages 15-19)	0.011 (0.003 ; 0.027)		
Children living with a single parent	Intercept	16.70 (15.62 ; 17.79)	23.95 (4.89)	13.32 (3.65)
	SES score, 1	-9.52 (-10.50 ; -8.55)		
	SES score, 2	-9.16 (-10.03; -8.29)		
	SES score, 3	-20.39 (-22.58 ; -18.19)		
	SES score, 4	-16.46 (-18.12 ; -14.80)		
	Year	0.35 (0.30 ; 0.40)		
	Population size (ages 0-17)	0.16 (0.14 ; 0.18)		
Reported and confirmed child abuse	Intercept	1.26 (1.19 ; 1.33)	0.11 (0.33)	0.11 (0.33)
	SES score, 1	-1.63 (-1.74 ; -1.52)		
	SES score, 2	-0.77 (-0.86 ; -0.69)		
	Year	0.017 (0.015 ; 0.019)		
	Population size (ages 0-17)	0.005 (0.003 ; 0.006)		
	Intercept	2.56 (2.37; 2.76)	1.15 (1.07)	0.11 (0.33)

Children with a handicap	SES score, 1	-2.32 (-2.65 ; -1.98)		
	SES score, 2	-1.17 (-1.41 ; -0.92)		
	Year	0.21 (0.20 ; 0.22)		
	Population size (ages 0-17)	0.005 (0.0002 ; 0.009)		
School drop-outs	Intercept	3.99 (3.79 ; 4.19)	0.86 (0.93)	0.49 (0.70)
	SES score, 1	-1.52 (-1.67 ; -1.38)		
	SES score, 2	-2.61 (-3.02 ; -2.20)		
	SES score, 3	-1.54 (-1.84 ; -1.25)		
	Year	-0.27 (-0.281 ; -0.269)		
	Population size (number of children in primary and secondary education)	0.020 (0.008 ; 0.032)		
Disadvantaged pupils	Intercept	9.57 (8.43 ; 10.99)	114.55 (10.70)	30.42 (5.52)
	SES score, 1	2.36 (1.50 ; 3.21)		
	SES score, 2	3.24 (2.27 ; 4.22)		
	SES score, 3	2.54 (1.60 ; 3.47)		
	SES score, 4	1.88 (0.91 ; 2.85)		
	SES score, 5	-1.75 (-2.75 ; -0.76)		
	SES score, 6	5.98 (3.88 ; 8.08)		
	SES score, 7	3.86 (1.89 ; 5.84)		
	Year	-1.46 (-1.49 ; -1.43)		
	Population size (number of children in	-0.21 (-0.27 ; -0.14)		

	primary education			
Children in special education	Intercept	2.75 (2.43 ; 3.06)	4.72 (0.2.17)	0.25 (0.50)
	SES score, 1	-2.17 (-2.72 ; -1.62)		
	SES score, 2	-0.78 (-1.16 ; -0.40)		
	Year	-0.0005 (-0.014 ; 0.013)		
	Population size (number of children in primary and secondary education)	0.06 (0.04 ; 0.07)		
Children participating in sport clubs	Intercept	24.16 (22.29 ; 26.04)	65.31 (8.08)	41.65 (6.45)
	SES score, 1	13.31 (12.04 ; 14.59)		
	SES score, 2	32.80 (29.28 ; 36.32)		
	SES score, 3	20.94 (18.59 ; 23.29)		
	Year	1.54 (1.38 ; 1.70)		
	Population size (ages 0-17)	-0.03 (-0.07 ; 0.01)		

SES score 1: first cubic spline, SES score 2: second cubic spline, SES score 3: third cubic spline, etc.

Supplementary Table S3. Characteristics of children in the age group 0-2 years old, n = 3,541.

**Variable**	**Median**	**95% range**	**Min-max**
SES score 2014	0.15	-2.84 – 1.90	-8.19 – 2.93
Children living in families on welfare in 2014 (%)	2.22	0.00 – 18.18	0.00 – 42.86
Child social services involved in 2015 (%)	0.79	0.00 – 3.75	0.00 – 40.00
Single parents in 2015 (%)	5.71	0.00 – 25.00	0.00 – 66.67

Reported and confirmed child abuse in 2015 (%)	0.00	0.00 – 3.33	0.00 – 11.25
Children with a handicap in 2015 (%)	0.83	0.00 – 5.00	0.00 – 14.00

Supplementary Table S4. Linear regression analyses for SES score and social indicators of child wellbeing, adjusted for the number of children living in a certain neighbourhood, n=3,541.

**Year 2014**	**0-2 year old children**		**0-17 year old children**	
**Variable**	**Beta estimate**	**95% CI**	**Estimate**	**95% CI**
Reported and confirmed child abuse*	1: **-3.61**<br>2: **-2.64**	(-4.57 ; -2.56)<br>(-2.96 ; -2.32)	1: **-2.27**<br>2: **-2.96**<br>**3: -2.67**<br>**4: -3.06**<br>**5: -2.35**<br>**6: -5.46**<br>**7: -2.41**	(-2.72 ; -1.78)<br>(-3.48 ; -2.39)<br>(-3.17 ; -2.13)<br>(-3.58 ; -2.50)<br>(-2.83 ; -1.86)<br>(-6.57 ; -4.25)<br>(-3.56 ; -1.57)
**Year 2015**	**0-2 year old children**		**0-17 year old children**	
**Variable**	**Estimate**	**95% CI**	**Estimate**	**95% CI**
Children living in families on welfare*	1: **-2.65**<br>2: **-3.18**<br>3: **-4.26**	(-2.81 ; -2.49)<br>(-3.97 ; -2.37)<br>(-4.78 ; -3.76)	1: **-2.97**<br>2: **-3.48**<br>3: **-4.25**	(-4.52 ; -3.59)<br>(-5.04 ; -4.66)<br>(-5.04 ; -4.66)

Child social services involved*	1: **-2.02**<br>2: **-5.25**<br>3: **-1.74**	(-2.26 ; -1.78)<br>(-6.19 ; -4.23)<br>(-2.18; -1.32)	1: **-0.90**<br>2: **-0.66**	(-1.29 ; -0.05)<br>(-0.74 ; -0.58)<br>(-1.56 ; -1.08)
Single parents*	1 **-1.06**<br>2: **-1.46**<br>3: **-1.31**<br>4: **-1.82**	(-1.44 ; -0.64)<br>(-1.73 ; -1.18)<br>(-2.20 ; -0.37)<br>(-2.22 ; -1.42)	1: **-0.90**<br>2: **-1.32**<br>3: **-1.01**<br>4: **-1.33**	(-1.21; -0.57)<br>(-1.53 ; - 1.11)<br>(-1.72 ; - 0.28)<br>(-1.62 ; - 1.06)
Children with a handicap*	1: 0.88<br>2: 0.41<br>3: 2.16<br>4: -0.23	(-0.34 ; 2.30)<br>(-0.32 ; 1.25)<br>(-0.48 ; 5.21)<br>(-1.00 ; 0.54)	1: **-0.49**<br>2: **-0.03**<br>3: **-0.65**	(-0.65 ; -0.31)<br>(-0.74 ; -0.70)<br>(-0.87 ; -0.43)

*cubic splines were applied to model for non-linearity

- estimates in bold are statistically significant alpha <0.05
